# Mutations in *EEA1* are associated with allergic bronchopulmonary aspergillosis and affect phagocytosis of *Aspergillus fumigatus* by human macrophages

**DOI:** 10.1371/journal.pone.0185706

**Published:** 2018-03-16

**Authors:** Nicola L. D. Overton, Axel A. Brakhage, Andreas Thywißen, David W. Denning, Paul Bowyer

**Affiliations:** 1 Manchester Fungal Infection Group (MFIG), Division of Infection, Immunity and Respiratory Medicine, School of Biological Sciences, Faculty of Biology, Medicine and Health, University of Manchester, Manchester Academic Health Science Centre, Manchester, United Kingdom; 2 Department of Molecular and Applied Microbiology, Leibniz Institute for Natural Product Research and Infection Biology (HKI), Jena, Germany; 3 Department of Microbiology and Molecular Biology, Institute of Microbiology, Friedrich Schiller University, Jena, Germany; 4 The National Aspergillosis Centre, University Hospital of South Manchester NHS Foundation Trust, Manchester Academic Health Science Centre, Manchester, United Kingdom; Woosuk University, REPUBLIC OF KOREA

## Abstract

Allergic bronchopulmonary aspergillosis (ABPA) in asthma is a severe, life-affecting disease that potentially affects over 4.8 million people globally. In the UK, ABPA is predominantly caused by the fungus *Aspergillus fumigatus*. Phagocytosis is important in clearance of this fungus, and Early Endosome Antigen 1 (*EEA1*) has been demonstrated to be involved in phagocytosis of fungi. We sought to investigate the role of *EEA1* mutations and phagocytosis in ABPA. We used exome sequencing to identify variants in *EEA1* associated with ABPA. We then cultured monocyte-derived macrophages (MDMs) from 17 ABPA subjects with *A*. *fumigatus* conidia, and analyzed phagocytosis and phagolysosome acidification in relation to the presence of these variants. We found that variants in *EEA1* were associated with ABPA and with the rate of phagocytosis of *A*. *fumigatus* conidia and the acidification of phagolysosomes. MDMs from ABPA subjects carrying the disease associated genotype showed increased acidification and phagocytosis compared to those from ABPA subjects carrying the non-associated genotypes or healthy controls.The identification of ABPA-associated variants in EEA that have functional effects on MDM phagocytosis and phagolysosome acidification of *A*. *fumigatus* conidia revolutionizes our understanding of susceptibility to this disease, which may in future benefit patients by earlier identification or improved treatments. We suggest that the increased phagocytosis and acidification observed demonstrates an over-active MDM profile in these patients, resulting in an exaggerated cellular response to the presence of *A*. *fumigatus* in the airways.

## Introduction

*Aspergillus fumigatus* is a ubiquitous environmental fungus. Humans inhale several hundred conidia each day, and these can persist in the lung [1–3]. The majority of overtly immunocompetent individuals clear *A*. *fumigatus* without infection or disease, however, some patients with asthma or cystic fibrosis (CF) develop allergic bronchopulmonary aspergillosis (ABPA) following inhalation and airway colonization with *A*. *fumigatus* [1, 4–6]. Patients suffer wheezing, expectoration of brown mucus plugs, have poorly controlled asthma or CF and develop ‘pneumonia’ [7]. They also display allergic responses, with highly elevated total blood IgE levels, and IgE reactivity to *A*. *fumigatus* either on skin prick test or with serum specific IgE [1, 4]. *A*. *fumigatus* is often isolated from patients’ sputum. Central bronchiectasis may develop, and mucoid impaction of bronchi with distal atelectasis may also occur. Untreated, ABPA can result in pulmonary fibrosis and respiratory failure [1, 4, 7]. If systematically sought, 1–8% of asthmatics seen in hospital referral clinics are found to have ABPA [8, 9]. In addition, up to 18% of adult CF patients also have ABPA [10]. It has been suggested that the global burden of ABPA with asthma potentially exceeds 4.8 million people [9].

It is unclear why certain asthmatics develop ABPA while the majority remain unaffected by exposure to *A*. *fumigatus*. It is thus very likely that there is an element of genetic susceptibility; ABPA can be found in families, suggesting a common genetic basis with low penetrance [11, 12]. In an Indian case series, 5% of cases were found to be familial in nature [13]. Although these family studies could be influenced by shared environment, a genetic element is supported by studies that have identified genetic polymorphisms associated with the disease [13, 14].

The immune response to *A*. *fumigatus* involves many cell types, including macrophages and neutrophils. These are highly phagocytic innate immune cells that phagocytose and kill the fungus, produce a variety of chemotactic and proinflammatory cytokines, and orchestrate an immune response [3, 15, 16]. Macrophages and neutrophils display different responses to different fungal morphologies [17] but as macrophages are present in the airways they may be the first innate immune cell to contact the inhaled fungus. Expression of immune genes by monocyte-derived macrophages (MDMs) exposed to *A*. *fumigatus* is different in MDMs from ABPA subjects compared to those from asthmatic subjects, which suggests that differing macrophage responses may be important in susceptibility to ABPA [18].

Phagocytosis is the mechanism by which relatively large (>0.5 *μ*m) particles, such as pathogens (bacteria, fungi etc.), dead cells or other objects such as polystyrene beads, are internalized [19–22]. It is an important tool in the macrophage armory and is an extremely complex process, involving many different genes and processes, such as actin formation and FcγR signaling [23–25]. Early Endosome Antigen 1 (*EEA1*) is a protein responsible for vesicle budding, transporting, tethering, and docking events in early endosomes, and has also been demonstrated to be involved in phagocytosis of fungi [26]. We investigated this gene in relation to ABPA and to phagocytosis of *A*. *fumigatus*.

## Materials and methods

### Ethics statement

Local Research Ethics Committees (South Manchester Research Ethics Committee and NRES Committee North West—Haydock) approved the study and all subjects gave written informed consent. REC references: 02/SM/286, 08/H1003/45 and 15/NW.0409.

### Subjects for exome sequencing

ABPA patients and atopic asthmatic controls without fungal sensitization were defined according to the diagnostic criteria in S1 Table. Subjects with ABPA were recruited from the tertiary referral clinic at the National Aspergillosis Centre (University Hospital of South Manchester [UHSM], UK) from March 2006 to August 2010. The Local Research Ethics Committee (South Manchester Research Ethics Committee, REC references: 02/SM/286 and 08/H1003/45) approved the study and all subjects gave written informed consent. Previously described asthmatic subjects were used as controls (16, 17). All subjects were adults and gave their own informed consent. No children participated in the study.

### Exome sequencing

DNA from 96 subjects with ABPA and 167 asthmatic controls was exome sequenced at the Centro Nacional de Análisis Genómico (CNAG) in Barcelona. 1μg of DNA was fragmented on a Covaris instrument to result in fragments of 150-300bp, and checked for quality control using the Bioanalyzer instrument. The entire volume of Covaris fragmented material was then processed through a standard Illumina sample preparation protocol: end repair, adenylation, ligation of adapters. After adaptor ligation and purification the library was amplified in the Pre-capture amplification step. A further QC was completed by the Bionalyzer to check library size (250–400 bp), quantify the pre-captured output material and confirm an appropriate size shift due to adaptor ligation (130bp shift). Forty five samples failed this QC (5 ABPA, 40 asthmatic) and were not used for exome sequencing. The remaining 218 samples (91 ABPA, 127 Asthma) were exome sequenced, using a Nimblegen 2.1 at a 6-plex, for a theoretical 50X coverage. Hybridization of baits, capture and post-capture amplification followed. QC at this stage involved an enrichment qPCR (pre-captured vs. post-captured material) and another bioanalayzer run to confirm the library size and concentration. Sequencing results were transferred to Genestack for analysis.

Data for 218 (91 ABPA, 127 asthmatic) individuals was analyzed by Genestack. The minimal underlying data set necessary for replication of the study are included in the paper and its Supporting Information files. Paired ended reads were aligned to the human genome GRCh37.71 using BWA 0.7.5a, and marked for duplicates using Picard 1.106. Variants were called using SAMtools 1.1 and BCFtools 1.1 (only keeping high quality variants with Quality > 20, MQ > 20, DP > 50, and including non-variant intervals). Effect, functional class and impact were predicted by SNPEff. This identified 5,642,881 variants (4,815,081 SNPs) and 898,105 Monomorphic Regions (MRs). Mutations in the *EEA1* gene were identified and analyzed for association with ABPA. Association analysis was completed using Genestack’s in-house application and allele frequencies in the ABPA and asthma groups were compared using the Fishers Exact Test and p<0.05 was considered significant. Only mutations with call rates of >90% in both the asthma and ABPA populations were analyzed.

### Subjects for phagocytosis experiments

For the phagocytosis experiments, 17 of the exome-sequenced ABPA subjects with different *EEA1* genotypes were recruited from the National Aspergillosis Centre (NAC) at the University Hospital of South Manchester. Subjects gave written informed consent and were recruited into the Manchester Allergy, Respiratory and Thoracic Surgery (ManARTS) Biobank, which has been approved by the Local Research Ethics Committee (NRES Committee North West—Haydock, ethics approval 15/NW.0409). ManARTS Biobank recruited subjects donated up to 100ml of blood, which was collected in heparinized blood tubes. In addition, buffy coat donated by healthy subjects was received from the NHS Blood and Transplant Service (NHSBT) for use in the phagocytosis experiments.

### Confirmation of EEA1 insertion-deletion mutation

For confirmation of the insertion-deletion mutation by Sanger sequencing, the 17 subjects in the phagocytosis study were re-sequenced by Eurofins Genomics (Ebersberg, Germany). Amplicons containing the *EEA1* indel were PCR amplified using the following cycling conditions: 95°C for 5min, 35 cycles of 95°C for 1 min, 63°C for 1 min and 72°C for 1 min, then 72°C for 2 min. PCR amplification was completed using Q5 Hot Start High-Fidelity 2X Master Mix (Ipswich, MA, USA) and 25ng of DNA and 2.5pmol of each primer (S2 Table) in a 12.5μL reaction. The amplicons were purified using the Agencourt AMPure XP beads (Beckman Coulter, High Wycombe, UK) and the protocol recommended by Illumina.

### Sequencing of the EEA1 insertion-deletion mutation in a replication population

For replication sequencing of the insertion-deletion mutation by MiSeq, a new population of 96 ABPA subjects and 96 asthmatic subjects (S1 Table) were selected from the Manchester Allergy, Respiratory and Thoracic Surgery (ManARTS) Biobank, which has been approved by the Local Research Ethics Committee (LREC, ethics approval 15/NW.0409). These subjects had been recruited from the UHSM previously, and had given written informed consent. Amplicons containing the *EEA1* indel were PCR amplified and purified as described for the confirmation PCR. Indexing PCR was then completed using the Nextera XT Index kit (Illumina, San Diego, CA, USA) and Kapa HiFi Hot Start polymerase (Kapa Biosystems, Cape Town, South Africa) and indexed samples were purified as previously. The concentration of each sample was measured and normalized to 10nM in Tris (pH8). Samples were then pooled equally to result in two pools containing the PCR amplicons of 96 individuals each. Samples were sequenced on an Illumina MiSeq sequencer (Illumina) by the University of Manchester Genomic Technologies Core Facility and data analysis was completed by the Bioinformatics Core Facility. Reads were adapter and quality trimmed using trimmomatic [27] and then mapped to the genome assembly (hg19) using bowtie2 [28]. Variant calling was performed using samtools 1.2 [29, 30] and bcftools 1.2 [29, 30].

### Generation of MDMs

MDMs were generated as described previously [31]. Briefly, PBMCs were extracted using a Ficoll-paque Plus (GE Lifesciences, Buckingham, UK) density gradient and stored in liquid nitrogen until use. These were then plated onto 24-well plates (2 × 10^6^/well) and monocytes were selected using 1.5 h plastic adherence. MDMs were then generated using GM-CSF and 15 days incubation. For phagocytosis experiments, sterilized 16mm diameter round glass cover slips (0.13–0.17mm thick) were placed inside the wells prior to addition of PBMCs, such that the MDMs were cultured onto the cover slips rather than the base of the wells.

### *A*. *fumigatus* strains and growth conditions

*A*. *fumigatus* wild-type strain (A1160) was cultivated on Sabouraud Dextrose agar flasks at 37°C for 3 days. Conidia were harvested in sterile PBS-Tween (0.01% [v/v] Tween-20) and filtered through glass wool to remove any hyphal fragments. Conidia concentration was determined using a haemocytometer.

### Quantification of phagocytosis

1x10^6^ *A*. *fumigatus* conidia were collected in 1ml sterile PBS-Tween (0.1% [v/v] Tween-20) and FITC-labeling of conidia was performed by addition of 5ml of 0.1 mg/ml FITC (F7250, Sigma-Aldrich) in 0.1M Na_2_CO_3_ at room temperature for 30 min. Labeled conidia were washed three times with 5ml PBS-Tween before being diluted to a concentration of 1x10^6^/ml in RPMI media. Media was removed from the MDM wells and 1ml (1x10^6^ conidia) was added. Plates were centrifuged for 5 minutes at 100g to synchronize the infection, and then transferred to 37°C, 5% (v/v) CO_2_ to start co-culture for 70 minutes. After this, the media was removed and the reaction was stopped by using ice-cold PBS. Labeling of extracellular conidia was performed by incubation with 250μg/ml Calcofluor White solution (in PBS) (CFW, Fluorescent Brightener 28, F3543, Sigma-Aldrich) for 30 minutes at room temperature. Wells were then washed three times with PBS before being fixed with 3% (v/v) formaldehyde for 15 minutes and washed again. Finally, MDMs were labeled by incubation with 10μg/ml solution of Concanavalin A Alexa Fluor 647 Conjugate (C21421, Molecular probes, Life technologies) (in PBS) for 30 minutes and washed again. The glass cover slips were then removed from the wells and inverted onto microscope slides for microscopy. Confocal imaging of different areas of the cover slips was completed using a Leica TCS Sp8 X confocal microscope (Leica Microsystems, Wetzlar, Germany). Excitation and emission wavelengths were as follows. Excitation at 405 nm and emission at 411–448 nm to visualize CFW fluorescence, excitation at 495 nm and emission at 501–552 nm to visualize FITC fluorescence, and excitation at 631 nm and emission at 649–726 nm to visualize Concanavalin A fluorescence. Simultaneous brightfield images were captured. Imaging was carried out at room temperature. Confocal images were captured using the Leica Application Suite X (1.8.0.13370) software (Leica Microsystems, Wetzlar, Germany). Images were analyzed to quantify phagocytosis. Internal conidia were labeled with FITC only, while external conidia were labeled with both FITC and CFW. MDMs were labeled with Concanavalin A. Only conidia in close proximity to MDMs were analyzed. Rates of phagocytosis were compared using t-tests.

### Quantification of phagolysosome acidification

Methods are similar to those described previously [32]. Prior to infection, MDMs were preloaded with 50 nM LysoTracker Red DND-99 (Invitrogen, Darmstadt, Germany) in RPMI medium for 2 hours. Immediately prior to infection, *A*. *fumigatus* conidia were stained with 250 μg/ml CFW for 30 minutes and washed three times with PBS-Tween. Conidia were diluted to a concentration of 4x10^5^/ml in pre-warmed RPMI medium containing LysoTracker. Media was removed from the cells and replaced with 1ml of this media (containing 4x10^5^ conidia). Plates were centrifuged for 5 minutes at 100g to synchronize the infection, and then transferred to 37°C, 5% (v/v) CO_2_ to start co-culture for 140 minutes (4 hours for siRNA experiment). Phagocytosis was stopped and cells were washed using PBS, and samples were immediately subjected to microscopy and visualized using a Leica TCS Sp8 X confocal microscope (Leica Microsystems). Excitation and emission wavelengths were as follows. Excitation at 405 nm and emission at 430–550 nm to visualize CFW fluorescence, and excitation at 575 nm and emission at 591–624 nm to visualize LysoTracker. Simultaneous brightfield images were captured. Imaging was carried out a room temperature. Confocal images were captured using the Leica Application Suite X (1.8.0.13370) software (Leica Microsystems). Images were analyzed to quantify phagolysosome acidification. Only conidia in close proximity to MDMs were analyzed. Compartments in which the LysoTracker had been concentrated were considered acidified. The percentage of conidia residing within acidified compartments (percentage acidification) was analyzed.

### Analyzing effect of EEA1 variants on phagocytosis and phagolysosome acidification

MDMs from each subject were used for the phagocytosis and phagolysosome acidification experiments, with two or three technical replicates per subject. For the phagolysosome acidification experiments, the percentage of phagocytosis was compared between subjects and groups. For phagocytosis experiments, an internal control was employed, which was a sample from a single healthy donor (designated NC07), and the samples were normalized to the rate of phagocytosis in this sample. The percentage of phagocytosis was then compared between subjects and groups using Mann-Whitney tests.

### siRNA experiments

siRNA molecules for the *EEA1* gene were purchased from Ambion Life Technologies (siRNA ID s15969 and diluted to 20μM for use. MDMs from healthy subjects were treated on day 14. For each sample, 10μL of siRNA was added to 15μL of RPMI media (supplement-free) while 1.5μL of lipofectamine was added to 23.5μL of RPMI media (supplement-free). These were then mixed together and incubated for 15 minutes at room temperature. The media was removed from the MDM wells and 500μL of fresh media (including supplements) was added. The 50μL of siRNA mix was then added to the well and mixed well. Incubation of cells continued at 37oC with 5% (v/v) CO_2_ and gene expression was measured by qRT-PCR after 24 hours. This demonstrated that siRNA treatment resulted in up to a significant reduction in *EEA1* expression. For the phagocytosis and phagolysosome acidification experiments, MDMs from a single healthy blood donor were used and these were treated with siRNA 24 hours prior to the experiment. Three biological replicates were completed, each with two or three technical replicates.

## Results

### Mutations in EEA1 are associated with ABPA

Seven mutations in *EEA1* were associated with ABPA (Table 1, S1 Fig). Six of these were known SNPs and one was a novel indel. As the indel was novel, we confirmed its presence by Sanger sequencing and we also replicated the association in a new population of ABPA and asthma patients. In this replication population we found an alternative allele frequency of 0% (0/192) in the ABPA group and 6.67% (12/192) in the asthma group, and again found a significant association with ABPA (p = 0.0004).

**Table 1:** *EEA1* mutations associated with ABPA.

Locus	SNP	Alleles (ref / alt)*	Effect^#^	Alt allele freq in asthma	Alt allele freq in ABPA	ABPA v Asthma FET p-value
12:93202801	*rs7970286*	***C****/T*	UTR_3_PRIME SYNONYMOUS_CODING DOWNSTREAM	33.6% (84/250)	22.5% (41/182)	0.0135
12:93247876	*rs60712367*	*A/****G***	DOWNSTREAM INTRON UPSTREAM INTRON	33.2% (83/250)	43.9% (79/180)	0.0267
12:93278423	*rs10859387*	***A****/G*	INTRON EXON INTRON	62.8% (157/250)	52.2% (93/178)	0.0365
12:93247777	*novel*	***-*** */ TAA*	INTRON UPSTREAM DOWNSTREAM INTRON	62.2% (158/254)	52.2% (95/182)	0.0392
12:93252527	*rs2133299*	***A****/G*	INTRON EXON INTRON	62.2% (158/254)	51.6% (94/182)	0.0307
12:93253408	*rs2019146*	*A/****G***	INTRON DOWNSTREAM INTRON	33.1% (84/254)	42.9% (78/182)	0.0444
12:93278060	*rs1809871*	***C****/T*	INTRON EXON INTRON	61.8% (157/254)	52.2% (95/182)	0.0495

*The allele that is associated with ABPA is shown in bold underline.

^#^Different effects in different transcripts (see S1 Fig for details)

### Distribution of the disease alleles between individuals

Of the 17 subjects used for the phagocytosis experiments, the same four subjects were homozygous for the disease associated allele of rs60712367 (GG), rs2133299 (AA), rs2019146 (GG) and rs1809871 (CC). These same four subjects, in addition to one more, were also homozygous for the disease associated alleles of rs10859387 (AA) and the novel insertion-deletion mutation at 12:93247775 (—). The distributions of the two non-disease associated genotypes are different in the remaining subjects.

We then investigated the distribution of the disease alleles between individuals in the original exome sequencing population, and found that of the 44 subjects carrying at least one of rs60712367 GG, rs2133299 AA, rs2019146 GG, rs1809871 CC, rs10859387 AA and 12:93247775 —, 41% (18/44) carried all six of these genotypes, while 11% (5/44), 11% (5/44), 7% (3/44), 7% (3/44) and 23% (10/44) carried five, four, three, two or one of these genotypes, respectively. This suggests that these disease-associated genotypes are often inherited together. The CC genotype of rs7970286 is much more common (53/91, 58%), and often found without the others.

### Mutations in EEA1 may affect phagocytic ability of MDMs

**Fig 1:**
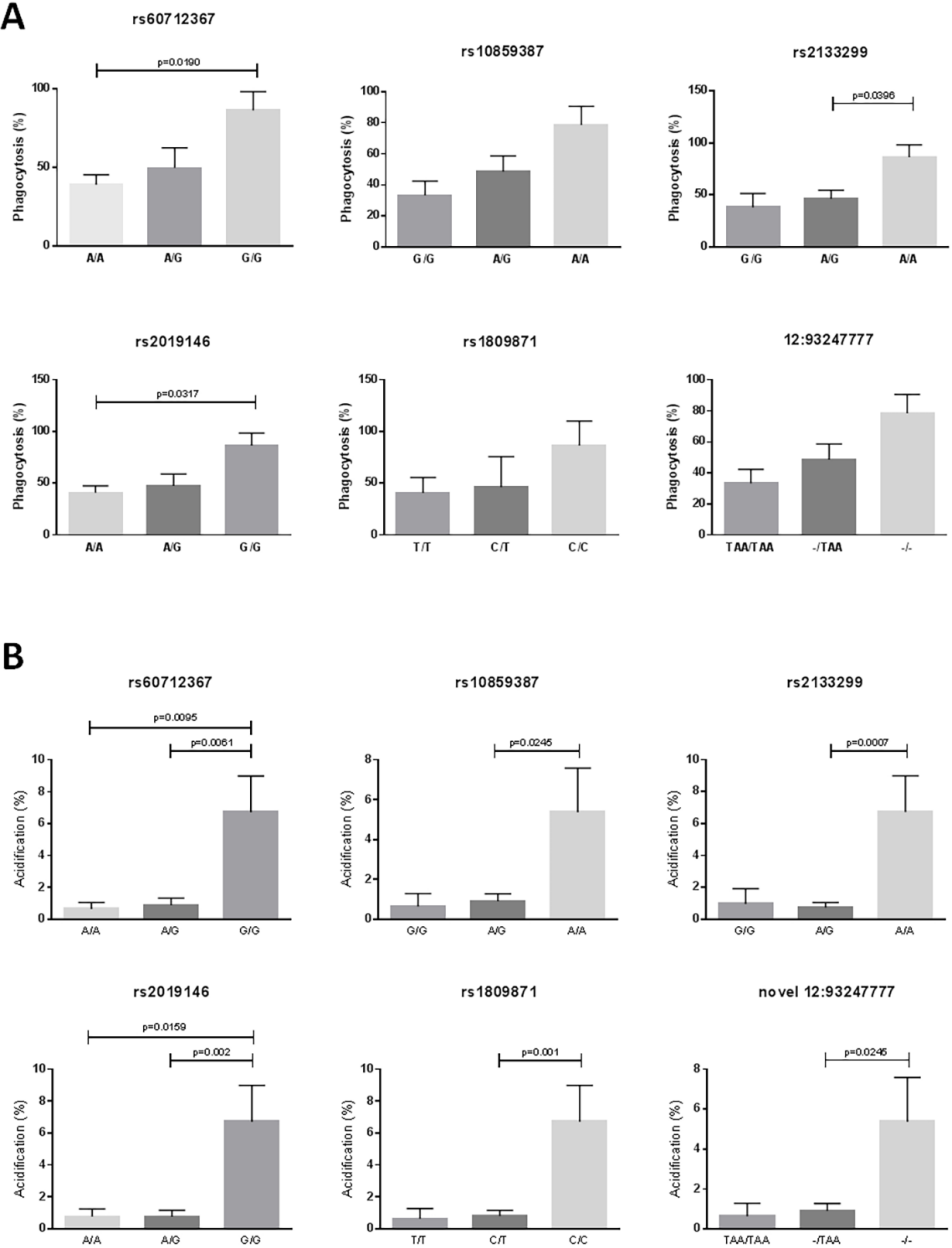
**Phagocytosis of *Aspergillus* conidia (A) and phagolysosome acidification as a result of this phagocytosis (B) in ABPA subjects with different genotypes**. Mean and SEM are shown, and groups are compared by Mann-Whitney tests.

**Fig 2:**
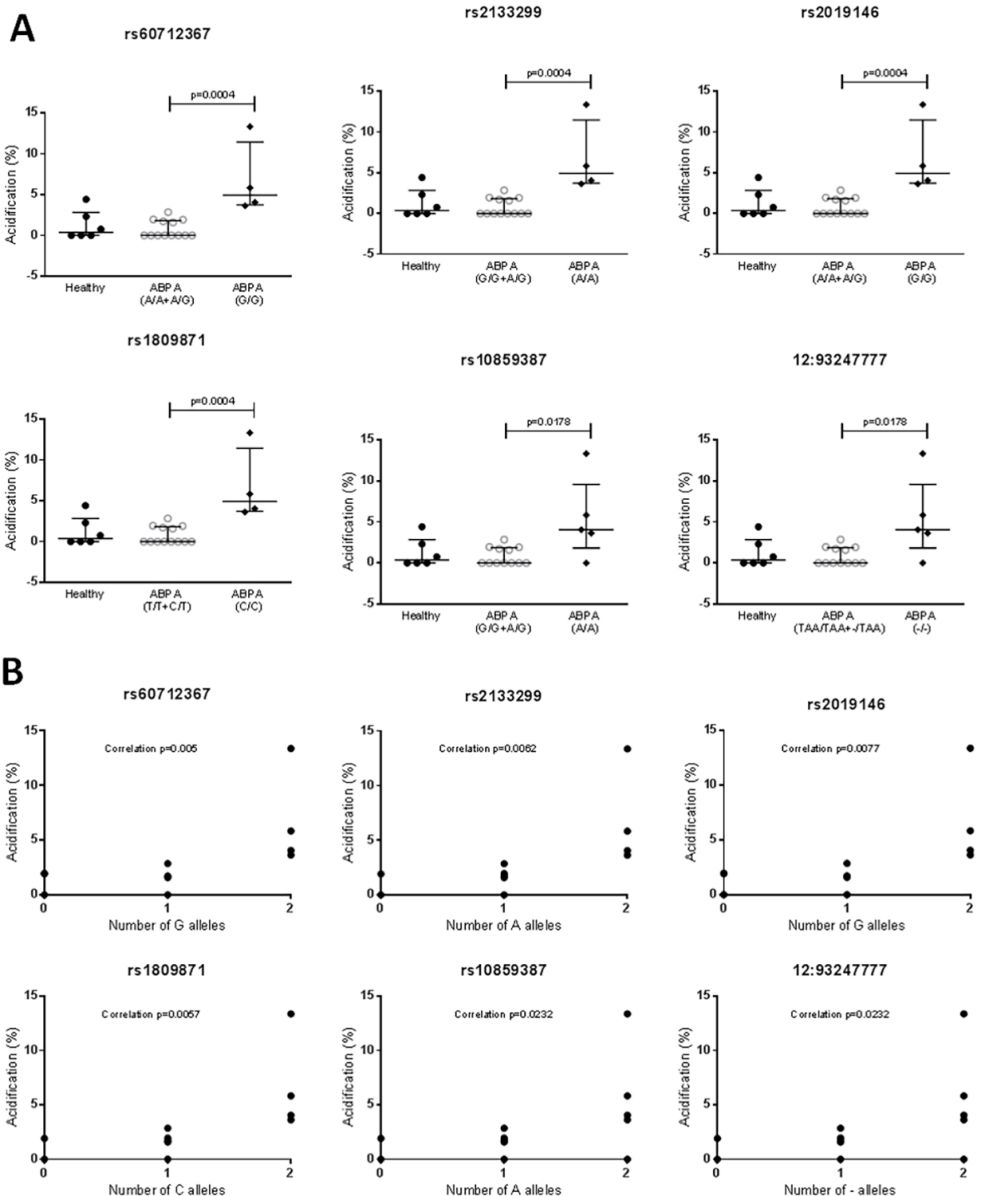
**Association of EEA1 genotype (A) and correlation of disease-associated alleles (B) with the percentage of phagolysosome acidification observed in MDMs after co-culture with *A*. *fumigatus*.** A) Median and interquartile ranges are shown. For the ABPA groups, each point represents an individual. For the healthy group, which is shown for interest, each point represents a replicate experiment using the same healthy subject. ABPA groups were compared by Mann-Whitney tests. Healthy groups were not compared as they only contained data for one individual, and are shown for interest only. B) Only ABPA subjects are shown, and each point represents an individual. Correlation was calculated using the Pearson R test.

**Fig 3:**
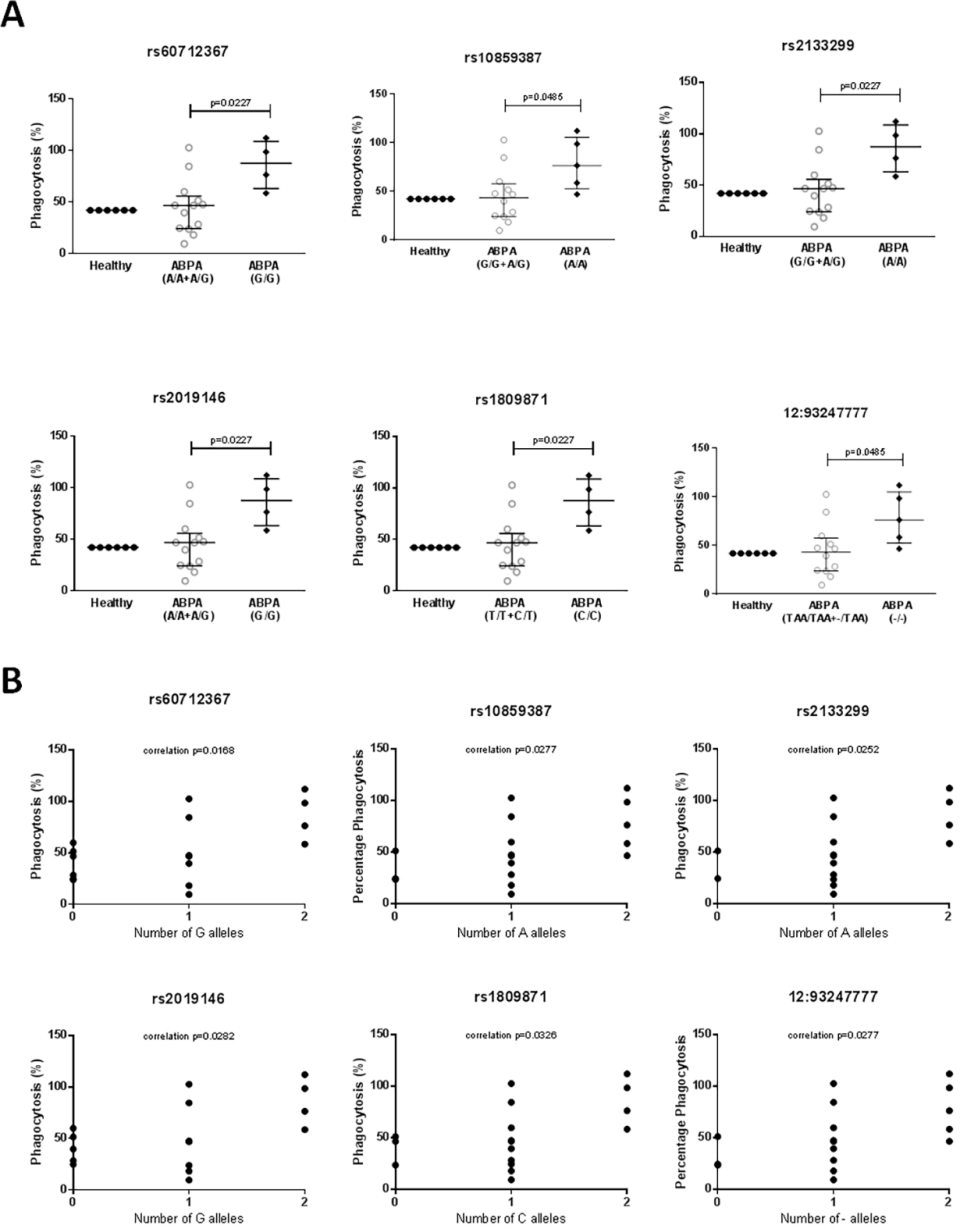
**Association of EEA1 genotype (A) and correlation of disease-associated alleles (B) with the percentage of conidial phagocytosis observed in MDMs after co-culture with *A*. *fumigatus*.** A) Median and interquartile ranges are shown. For the ABPA groups, each point represents an individual. For the healthy group, which is shown for interest, each point represents a replicate experiment using the same healthy subject. ABPA groups were compared by Mann-Whitney tests. Healthy groups were not compared as they only contained data for one individual, and are shown for interest only. B) Only ABPA subjects are shown, and each point represents an individual. Correlation was calculated using the Pearson R test.

MDMs from subjects with different genotypes showed differences in the levels of phagocytosis of *Aspergillus* conidia and phagolysosome acidification as a result of this phagocytosis (Fig 1). The MDMs from subjects carrying the rs60712367 GG, rs2133299 AA, rs2019146 GG, rs1809871 CC, rs10859387 AA and 12:93247777—genotypes showed higher levels of both phagocytosis and phagolysosome acidification than those from subjects carrying the GA/AA, AG/GG, GA/AA, CT/TT, AG/GG and–TAA/TAATAA genotypes respectively and the number of disease-associated alleles were correlated with an increasing percentage of phagolysosome acidification (Figs 2 and 3). In addition, levels of acidification in the MDMs from the healthy controls used (assayed in various experiments) were similar to those in MDMs from subjects carrying the non-disease associated allele. As this data was from a single individual it was not analyzed statistically and is shown for interest only, but it suggests that acidification and phagocytosis are higher than normal in the ABPA subjects carrying the disease genotypes. There was no association between the rs7970286 genotype and phagolysosome acidification or phagocytosis, although a similar trend was seen, with MDMs from patients with the disease genotype (CC) displaying higher levels of both than those with the CT or TT genotypes (not significant, data not shown).

### siRNA knockdown of EEA1

**Fig 4:**
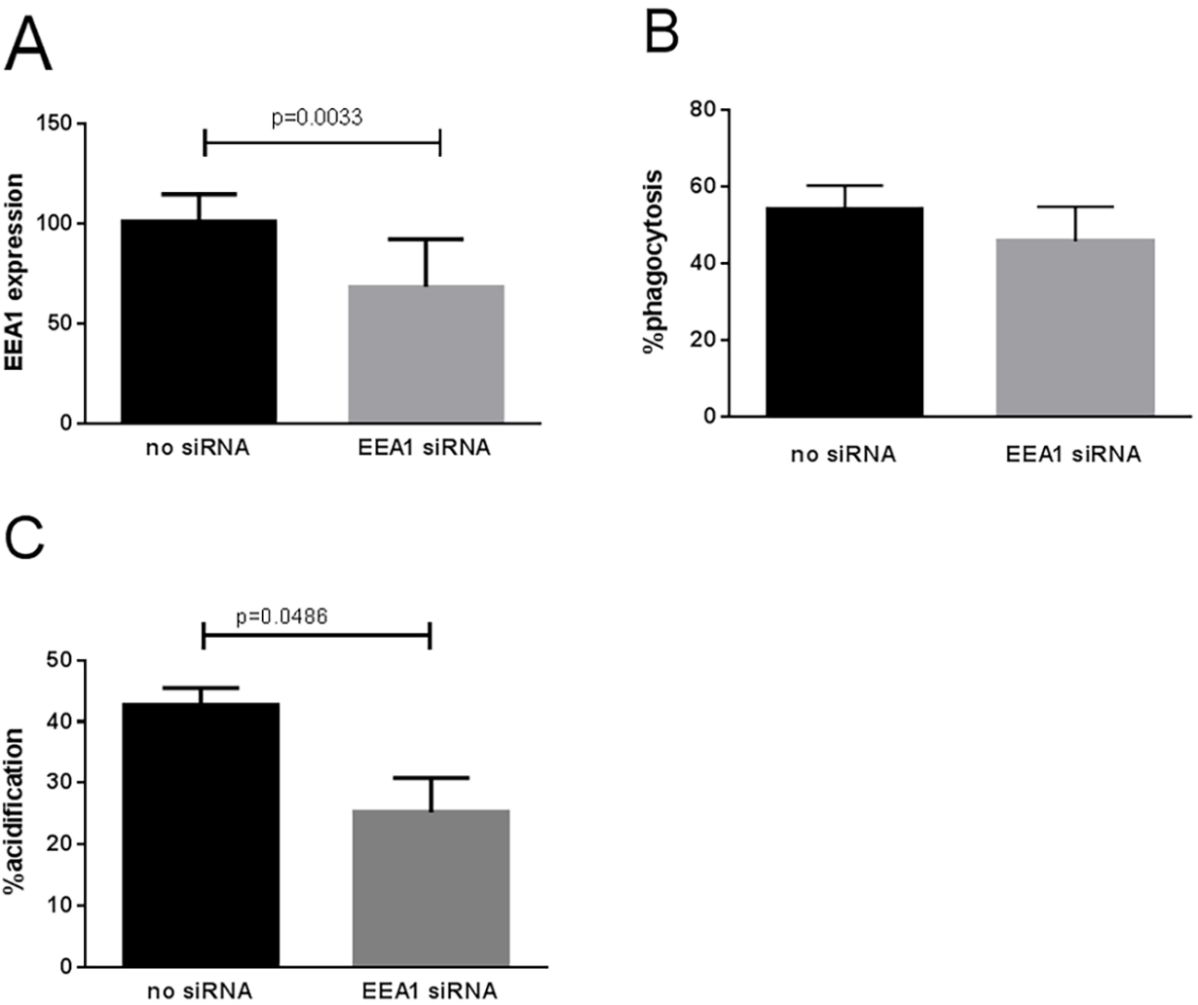
*EEA1* expression, phagocytosis and phagolysosome acidification after siRNA treatment. Mean and SEM shown. Groups compared by t-test. A) siRNA treatment. B) Phagocytosis. C) Phagolysosome acidification.

siRNA knockdown of *EEA1* significantly reduced gene expression by 32% (p<0.001,Fig 4). However, this *EEA1* only slightly reduced the phagocytic ability of MDMs from healthy donors (46% vs. 54%), and this was not statistically significant (Fig 4). This may be due to many contributory proteins affecting the rate of phagocytosis combined with the modest (but significant) reduction in gene expression at 32%. Despite this, a significant reduction in phagolysosome acidification was observed in the siRNA treated MDMs (25% vs. 43%, p = 0.0486) (Fig 4).

## Conclusions

Phagocytosis by macrophages is an important factor in the host response to *A*. *fumigatus*. It is also an extremely complex process, involving many different stages and processes, such as actin formation and FcγR signaling [23–25]. Cell membrane receptors first recognize antibodies on the target, causing initial cup formation, followed by the extension of membrane protrusions called pseudopodia to surround this target, and closure of the target within the cell by a zipper-like mechanism. This is followed by fusion with lysosomes, acidification of the phagolysosome, superoxide and hydroxyl radical generation and degradation of the target [19–21, 23–25, 32, 33]. All of these processes require control and each involves different signaling and effector molecules and proteins, all of which could be affected by mutations within the genes for these. Studies suggest that over 250 genes are involved in phagocytosis [24, 34], and as such, this is a difficult process to study and dramatic effects of single genes are unlikely. However, despite this, we have attempted to investigate phagocytosis in ABPA.

Here, we have shown that the phagocytic ability of MDMs varies considerably between individuals, and may be linked to variants in the *EEA1* gene. We have shown that seven variants (six SNPs and one indel) in the *EEA1* gene are independently associated with ABPA and that six of these are also associated with differences in the phagocytic ability of MDMs. We have found that the homozygotes of the disease-associated allele are often found together within the same subject, suggesting they are inherited together. This is confirmed by the linkage disequilibrium (LD) found in the 1000 genomes project (as viewed on Ensembl). LD was not determined for rs7970286 as this is a long distance from the other *EEA1* SNPs, but the other known SNPs are all in high LD with each other (r^2^>0.80). We have also shown that knockdown of *EEA1* by siRNA can affect the phagocytic ability of previously healthy MDMs.

The rs60712367 G allele, rs2133299 A allele, rs2019146 G allele and rs1809871 C allele, rs10859387 A allele and the novel indel at 12:93247775—allele were all associated with ABPA disease and with increasing levels of both phagocytosis and phagolysosome acidification. Levels of phagocytosis and acidification in the one healthy control assayed (assayed in various experiments) appeared similar to those in subjects carrying the non-disease associated genotypes. As this is data from only one subject, few conclusions can be drawn, but it may suggest that the lower levels of phagocytosis and acidification observed with the non-disease genotypes are normal, and that the higher levels seen in the samples with the disease-associated genotypes are an over-active response.

*EEA1* is a protein responsible for vesicle budding, transporting, tethering, and docking events in early endosomes, and has been demonstrated to be involved in phagocytosis of fungi [26]. In murine macrophages infected with *Talaromyces* (*Penicillium*) *marneffeii*, labeling of phagosomes for *EEA1* is observed within 30 min of heat-killed conidia internalization, and the percentage of *EEA1* that associates with live conidia has been shown to be significantly lower than that with heat-killed conidia [26]. Similarly, when expression of *EEA1* was measured in murine macrophages infected with *Paracoccidioides brasiliensis*, *EEA1* protein was present in the early endosome, in the first hours of infection, but strongly decreased after the fungus was internalized. The authors suggest that alterations in phagosome maturation caused by *P*. *brasiliensis* originate upstream of the *EEA1*-mediated trafficking and that the exclusion of *EEA1* leads to a block in trafficking from the trans-Golgi network to phagosomes [35].

Mutagenesis of amino acid positions between 39 and 60 in the *EEA1* protein reduces the interaction of *EEA1* with RAB5C [36], which is important in regulation of endocytosis and fusion of endosomes [37], and *EEA1* has been shown to be a Rab5 effector that is required for endosome fusion [38]. As such, loss or gain of *EEA1* function could affect the rate of fusions of vesicles to endosomes or phagolysosomes, which could in turn affect release of vesicle contents and subsequent acidification. Previous studies have suggested that maturation of *A*. *fumigatus* phagosomes requires fusion with the compartments of the endocytic pathway and that killing of *A*. *fumigatus* conidia depends on phagolysosome acidification [39].

Mutagenesis of C-terminal positions (specific positions between AA 1349 and 1405) reduces phosphatidylinositol 3-phosphate binding and localization of *EEA1* to endosomes [40, 41]. *EEA1* contains two zinc fingers (www.uniprot.org/uniprot/Q15075); a C2H2-type at position 41–64, and a FYVE-type at position 1352–1410. The mutations we have identified do not fall within the same exon as either of the zinc finger domains or the RAB5C or PI3P binding domains, but may affect the function or expression of *EEA1* in other ways, possibly by affecting transcript processing and hence protein level, or by more directly affecting regulation of the protein level.

Our work provides evidence to support a role for phagocytosis defects in the development of ABPA, and although previous work suggests that *EEA1* is primarily involved in endosome fusion to the phagosome, rather than to particle engulfment and internalization, our results support a role for *EEA1* in both phagocytosis and phagolysosome acidification of *A*. *fumigatus*, as both of these processes appear affected by the presence of *EEA1* SNPs. Unlike other SNPs in EEA1, the SNPs identified in this study have not been investigated previously, and we do not know how they may affect the function of EEA1. Further study is required to investigate this further. The introduction of mutations into healthy cells, or the repair of mutations in disease cells, by a method such as CRISPR could be useful.

It seems from our results that ABPA patients carrying the disease associated *EEA1* alleles have significantly increased phagocytosis and phagolysosome acidification, and further functional and mechanistic experimentation, which were outside the remit of this initial study, will be useful to elucidate a mechanism for how the mutations result in this increased phagocytosis, and why this is detrimental in ABPA. It will be interesting to know if the increased phagocytosis and acidification is associated with increased killing of Aspergillus or alters the inflammatory response. We hypothesize that the observed increased phagocytosis and acidification demonstrates an over-active MDM profile in these patients. We suggest that the increased phagocytosis and acidification leads to increased killing and increased antigen presentation by the MDMs, which, combined with the over-active MDMs, results in an exaggerated cellular response to the presence of *A*. *fumigatus* in the airways.

We acknowledge that we have studied only 17 patients in this study. Despite this, we believe our work provides an interesting insight into the role of phagocytosis in ABPA. We believe that phagocytosis will be one of many processes that are defective in ABPA subjects and that only a subset of ABPA disease will be related to phagocytosis. Other patients are likely to have other immune deficiencies that affect their ability to clear the fungus.

The strong associations of the *EEA1* mutations we have identified with ABPA compared with control asthmatics, combined with our biological findings from these same patients, clearly indicate that defects in phagocytosis are an important aspect of the pathogenesis of ABPA. Further identification and functional validation of these variants and genetic defects in patients will revolutionize our understanding of susceptibility to ABPA and other fungal and infectious diseases. Our work may lead to development of robust diagnostic tests for the at-risk population, as well as suggesting novel areas for research into drug targets for treatments. This would be of real benefit to patients who are affected by this disease.

## Supporting information

S1 Table Diagnostic criteria for ABPA patients and asthmatic controls. (DOCX)

S2 Table Primers used for PCR amplification of *EEA1* insertion-deletion mutation. (DOCX)

S1 Fig Location of identified ABPA-associated mutations within the different *EEA1* transcripts. Image from Ensembl GRCh37 (http://grch37.ensembl.org), with mutations added as custom track by Ian Donaldson of the Bioinformatics core facility at the University of Manchester.
(DOCX)
