# Supplementary material for: Mutations in EEA1 are associated with allergic bronchopulmonary aspergillosis and affect phagocytosis of Aspergillus fumigatus by human macrophages
Source: PLoS One. 2018 Mar 16;13(3):e0185706. doi: 10.1371/journal.pone.0185706 (PMC5856258; doi:10.1371/journal.pone.0185706)
Supplement: S1 Table — (DOCX) [file pone.0185706.s001.docx]

Supplementary Table S1 - Diagnostic criteria for ABPA patients and asthmatic controls

| **Disease** | **Diagnostic Criteria** |
| --- | --- |
| **ABPA** | *All the following are required:*  Total serum IgE >1,000 IU/ml (at any time)  Either positive SPT for *Aspergillus* or *Aspergillus* specific IgE  Current or historical evidence of eosinophilia  *Further indicators:*  Almost all patients have asthma (or cystic fibrosis, n=3) and over 50% central bronchiectasis on CT, but these were not required for inclusion.  Either recurrent obstruction (mucoid impaction) or episodes coughing up plugs of thick mucus (containing hyphae and eosinophils)  Positive *Aspergillus* precipitins or raised *Aspergillus* IgG titer |
| **Atopic (non-fungally atopic) asthmatic** | *All the following are required:*  Physician diagnosed asthma  No diagnosis of aspergillosis  Negative SPT (at 3mm cut-off) and/or IgE (<0.4) to all fungi tested, including *Alternaria alternata*, *Candida albicans*, *Cladosporium herbarum*, *Penicillium notatum, Trichophyton mentagrophytes*, *A. fumigatus.*  Positive SPT (at 3mm cut-off) and/or IgE (<0.4) to any allergen non-fungal allergen tested (e.g. mite, cat, dog and grasses)  NOTE: Only SPT or IgE need be completed, but if both are done and one is positive this is classified as a positive result. |

SPT, Skin Prick Test.
