# Supplementary material for: Mutations in EEA1 are associated with allergic bronchopulmonary aspergillosis and affect phagocytosis of Aspergillus fumigatus by human macrophages
Source: PLoS One. 2018 Mar 16;13(3):e0185706. doi: 10.1371/journal.pone.0185706 (PMC5856258; doi:10.1371/journal.pone.0185706)
Supplement: S2 Table — (DOCX) [file pone.0185706.s002.docx]

Supplementary Table S2 - Primers used for PCR amplication of *EEA1* insertion-deletion mutation

| **Primer** | **Sequence (includes Illumina tag for MiSeq)** |
| --- | --- |
| **Forward** | TCGTCGGCAGCGTCAGATGTGTATAAGAGACAGAAATTCATTTGGCAGGCATC |
| **Reverse** | GTCTCGTGGGCTCGGAGATGTGTATAAGAGACAGTGCCTGCTGGTCTCTTACAG |
