# Supplementary material for: Mutations in EEA1 are associated with allergic bronchopulmonary aspergillosis and affect phagocytosis of Aspergillus fumigatus by human macrophages
Source: PLoS One. 2018 Mar 16;13(3):e0185706. doi: 10.1371/journal.pone.0185706 (PMC5856258; doi:10.1371/journal.pone.0185706)
Supplement: S1 Fig — Image from Ensembl GRCh37 (http://grch37.ensembl.org), with mutations added as custom track by Ian Donaldson of the Bioinformatics core facility at the University of Manchester. (DOCX) [file pone.0185706.s003.docx]

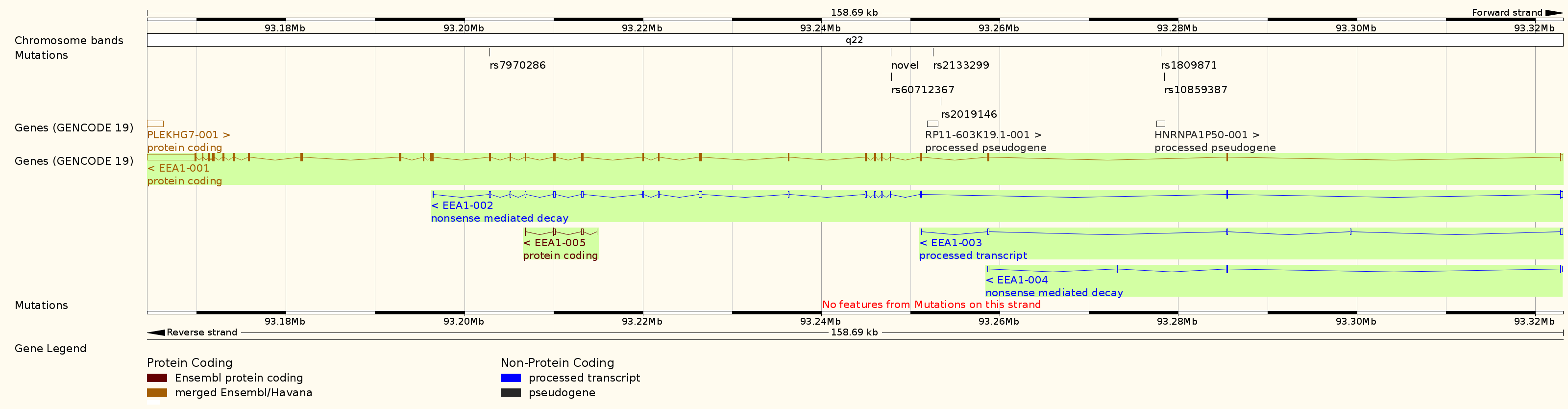


**Supplementary Figure S1 – Location of identified ABPA-associated mutations within the different *EEA1* transcripts.** Image from Ensembl GRCh37 (http://grch37.ensembl.org) [[42](#_ENREF_42)], with mutations added as custom track by Ian Donaldson of the Bioinformatics core facility at the University of Manchester.
